# Manipulation of phasic arousal by auditory cues is associated with subsequent changes in visual orienting to faces in infancy

**DOI:** 10.1038/s41598-023-49373-x

**Published:** 2023-12-12

**Authors:** Giorgia Bussu, Ana Maria Portugal, Lowe Wilsson, Johan Lundin Kleberg, Terje Falck-Ytter

**Affiliations:** 1 Development and Neurodiversity Lab, Department of Psychology, Uppsala University, Von Kraemers Alle 1C, 754 32 Uppsala, Sweden; 2 Department of Women’s and Children’s Health, Center of Neurodevelopmental Disorders (KIND), Centre for Psychiatry Research, Karolinska Institutet & Stockholm Health Care Services, Region Stockholm, Stockholm, Sweden; 3 Department of Clinical Neuroscience, Centre for Psychiatry Research, Karolinska Institutet & Stockholm Health Care Services, Region Stockholm, Stockholm, Sweden; 4 Department of Molecular Medicine and Surgery, Karolinska Institutet, Stockholm, Sweden

**Keywords:** Oculomotor system, Sensory processing, Human behaviour

## Abstract

This eye-tracking study investigated the effect of sound-induced arousal on social orienting under different auditory cue conditions in 5-month-old (n = 25; n = 13 males) and 10-month-old infants (n = 21; n = 14 males) participating in a spontaneous visual search task. Results showed: (1) larger pupil dilation discriminating between high and low volume (b = 0.02, p = 0.007), but not between social and non-social sounds (b = 0.004, p = 0.64); (2) faster visual orienting (b =  − 0.09, p < 0.001) and better social orienting at older age (b = 0.94, p < 0.001); (3) a fast habituation effect on social orienting after high-volume sounds (χ^2^(2) = 7.39, p = 0.025); (4) a quadratic association between baseline pupil size and target selection (b =  − 1.0, SE = 0.5, χ^2^(1) = 4.04, p = 0.045); (5) a positive linear association between pupil dilation and social orienting (b = 0.09, p = 0.039). Findings support adaptive gain theories of arousal, extending the link between phasic pupil dilation and task performance to spontaneous social orienting in infancy.

## Introduction

The ability to orient to salient stimuli develops very early in infancy and it influences the way an individual infant experiences the external world, with implications for learning opportunities, and in turn, later development. According to the adaptive gain theory (Aston-Jones and Cohen^1^), behavioural performance in tasks that require focussed attention is influenced by changes in arousal level. These changes reflect activity in the locus coeruleus-norephinephrine (LC-NE) system in interaction with top-down cortical influences acting largely through the basal forebrain-acetylcholine (BF-Ach) system^2^. In particular, phasic changes in LC activity, occurring about 100 ms after stimulus onset (Hayat et al.^3^)), are thought to facilitate perceptual performance^4^ and adaptive behavioural responses to the stimuli by increasing the signal-to-noise ratio via autoinhibition and input excitation^5^, promoting exploitation of the current focus of attention over exploration of the environment. Fluctuations in pupil size reflect changes in noradrenergic and acetylcholine neuromodulatory systems^6^; therefore, pupillometry can be used as an index of global arousal level and alertness to stimuli^7^.

Phasic changes in neuromodulatory activity (phasic arousal) have been previously investigated in adults through task-evoked changes in pupil diameter and related to task performance^8–10^. However, little is known about these associations early in infancy^11,12^. While it is not possible to instruct infants to perform a specific task, we used a spontaneous visual search task building on a well-known infant paradigm, the face pop-out paradigm^13,14^, as an ecologically valid solution to investigate influences of phasic arousal on visual orienting in infancy, whereby fast orienting to a target stimulus was used as infant equivalent to instructed task performance in adults^11^. In this task, one visual stimulus selected as target based on unique task-relevant features (e.g., faces for social content) is presented among distractors appearing simultaneously on the screen. This set-up exploits the general tendency among infants to orient to faces with higher likelihood compared to chance level^13,15^. Nevertheless, this tendency appears to change across early development, with an increasing preferential looking to faces after 6 months of age while physical salience seems to have a stronger influence on infants’ looking behaviour earlier in the first year of life^16,17^. Scores on the task (per trial or averaged for an individual) can then be conceptualized as ‘task performance’. In the specific framework of this study, the spontaneous visual search task allowed us to test the association between arousal level and performance in the form of selective attention to socially relevant stimuli (i.e., faces) early in infancy, which is a fundamental mechanism for social information processing and development of social skills^18^.

Previous work has investigated the modulatory effect of sound-induced arousal level on visual orienting abilities using an analogous task by measuring pupil dilation in 6.5-month-old infants^11^. The results showed different associations between pupil dilation response and speed of looking behaviour (indexed by latency of first look to any object in the visual array presented) versus selectivity of spontaneous attention (indexed by first look to the target; i.e., face). For latency, results showed a negative linear relation with pupil dilation; supporting the idea that higher phasic arousal is linked to faster orienting. For first look to the face, the authors found instead evidence for an inverted U-shaped relation with pupil dilation, suggesting best performance in terms of selectivity at intermediate levels of phasic arousal while the adaptive gain theory predicts such an association for tonic arousal. However, these findings need further replication and it remains a relevant question to understand how properties of the stimuli used to elicit phasic arousal may influence the observed association with visual orienting performance. While previous studies have examined the effect of auditory stimuli manipulations in older age groups^19^, further work is needed in infancy to understand how changes in stimulus properties may influence the pupil response and its association with visual orienting, or whether these associations may vary across development.

In the present study, we extended the work from Kleberg et al.^11^ to experimentally investigate the effect of alerting sounds on the association between pupil dilation and visual orienting to faces in 5- and 10-month-old infants. Age groups were selected to be close to the age investigated by Kleberg et al.^11^, yet respectively in the former and latter half of the first year of life, when developmental changes in the drivers of infants’ looking preference may occur^17^. Hypotheses and aims for this study were pre-registered (see https://osf.io/wds3g). In particular, this study aimed to investigate: (1) the effect (induced arousal and visual orienting) of sounds varying in social content and (2) volume level; (3) habituation effects in pupillary and gaze behaviour following repeated sounds; (4) developmental (i.e. age) effects on the modulation of phasic arousal and its relation with social orienting in infancy.

To this aim, we collected eye-tracking data from infants who took part in an audio-visual experiment based on a spontaneous visual search task, in which a picture of a face was presented among distractors. Visual stimulation was preceded by auditory cues manipulated along 3 dimensions: audio content (3 levels: silent, social, and non-social); audio volume (2 levels: low and high); and order (2 levels: first and second repetition). Based on previous work^11^, we hypothesized that social sounds would trigger larger pupil dilation and shorter latency of first look to any image in the visual array compared to non-social sounds or absence of auditory cue. The hypothesized effect of social sounds also aligns with developmental theories highlighting the role of infant-directed speech in capturing and sustaining infant attention^20,21^, possibly mediated by arousal. Furthermore, previous work has shown that pupil size is proportional to loudness, suggesting a link between sound deviance and induced arousal level^22,23^. Thus, we hypothesized that auditory stimuli at high volume levels would result in larger pupil dilation and faster first look latency compared to the low volume condition. Previous work with adults has shown a rapid habituation effect on pupillary response to repeated sounds^22^. Therefore, repetition of the same auditory stimulus in our study was expected to reduce the novelty effect of the auditory cue, resulting in a decrease in pupil dilation and longer latency of first look to any target compared to the first sound occurrence. We did not have a directional hypothesis in terms of modulation of target selection by auditory cue content and volume, or repetition. However, we hypothesized that larger pupil dilation would be negatively associated with latency of first look to any target and would show an inverted U-shaped relation with target selection (i.e., first look at the face), with optimal performance at intermediate levels of phasic arousal^11^. Finally, we expected to find developmental effects in visual orienting abilities; in particular, shorter latencies at 10 months compared to 5 months. However, we had no directional hypothesis about developmental effects on pupil dilation and its modulation by auditory alerting cues of different content, volume and repetition.

## Results

### Phasic arousal: pupil dilation

**Figure 1:**
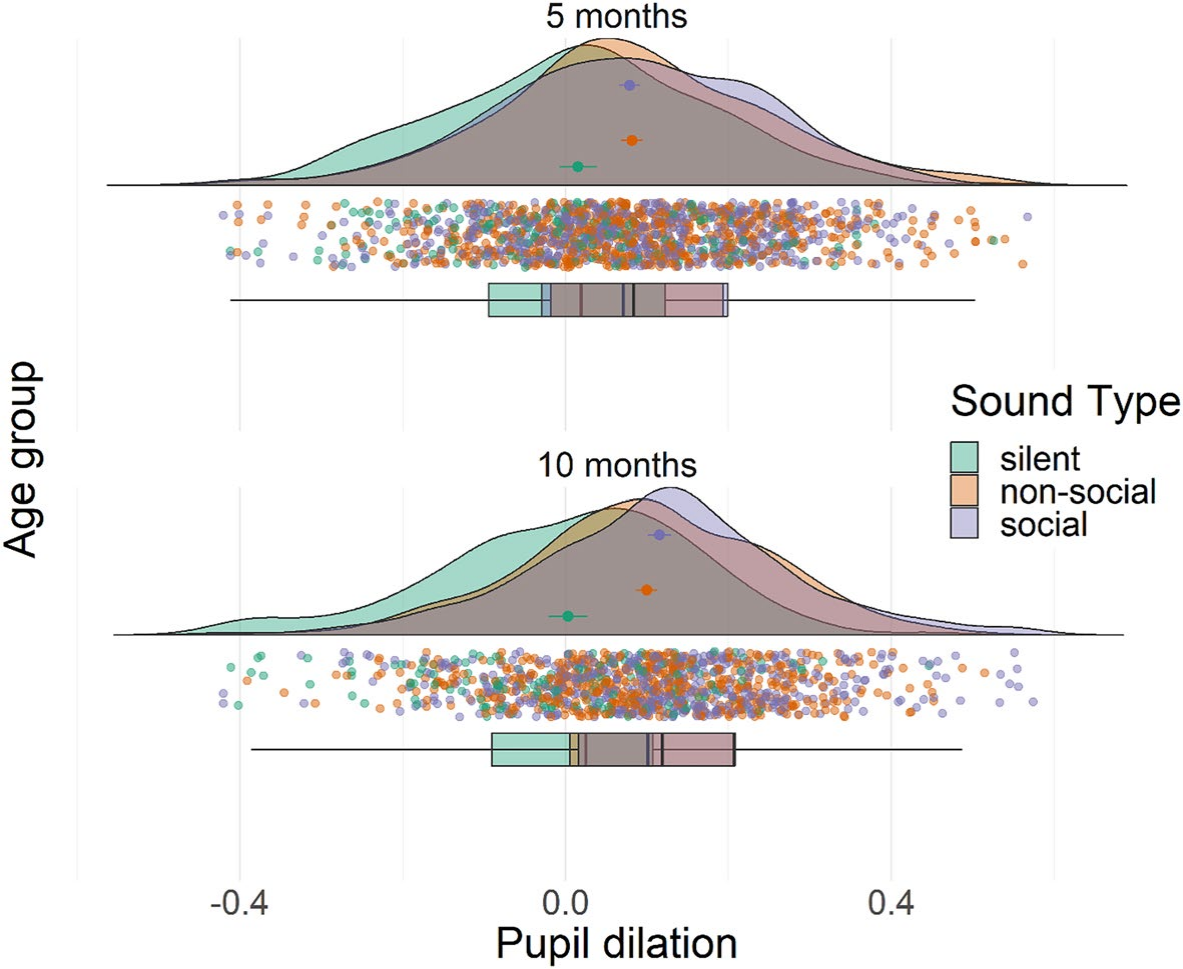
Effect of sound content (i.e., social, non-social, silent) on average pupil dilation by age (5 months: upper plot; 10 months: lower plot). Data points in the figure show individual trial data, while mean and standard error for each condition are shown in the clouds, and boxplots (with 25% to 75% whiskers) are reported below.

We found a significant main effect of sound content on pupil dilation (F(2, 2504) = 49.1, p < 0.001), whereby social (b = 0.07, p < 0.001) and non-social cues (b = 0.08, p < 0.001) induced larger pupil dilation compared to silent trials, while social and non-social cue conditions did not differ (b = 0.004, p = 0.64 for non-social vs. social sounds). However, the effect of sound content on pupil dilation (including the silent condition) was moderated by age, with a significant interaction effect between sound content and age group (F(2, 2504) = 3.74, p = 0.024) showing a larger effect of sound content (social vs. non-social vs. silent) at 10 months compared to 5 months. Specifically, follow-up tests on marginalized effects within age groups showed larger pupil dilation induced by social (b = 0.12, p < 0.001) and non-social sounds (b = 0.10, p < 0.001) compared to silent trials at 10 months compared to 5 months (respectively, b = 0.07, p < 0.001 for the social condition, and b = 0.08, p < 0.001 for the non-social condition), indicating a developmental effect of age on sound-induced pupil dilation. We found no significant age effect on specific sound conditions (social vs. non-social vs. silent), or any difference for non-social vs. social sounds at 5 (b = 0.004, p = 0.67) or 10 months (b =  − 0.01, p = 0.12), but descriptively, direction of the association changed from 5 to 10 months. Results are shown in Fig. 1.

**Figure 2:**
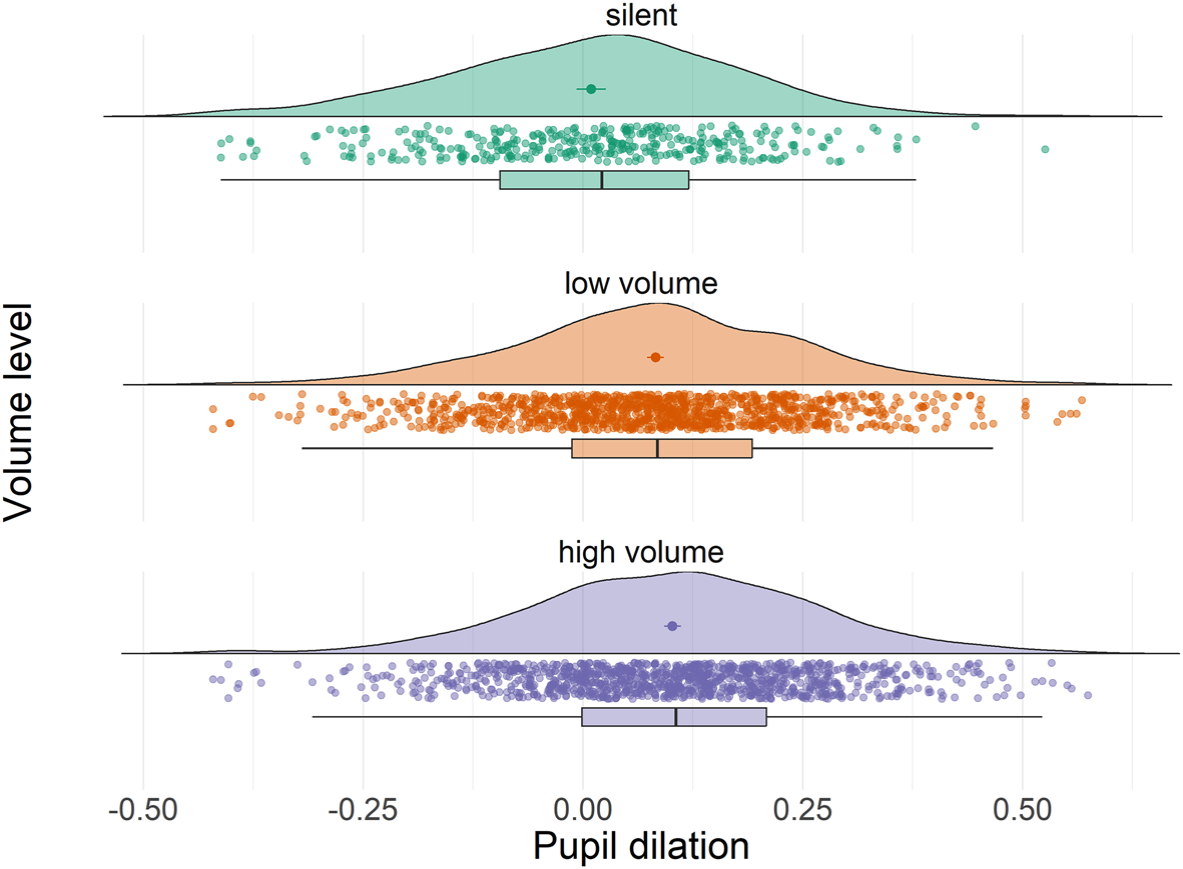
Effect of volume of auditory cue (i.e., high, low, silent) on average pupil dilation (standardized). Data points in the figure show individual trial data while mean and standard error for each condition are shown in the clouds, and boxplots (with 25% to 75% whiskers) are reported below.

We also found a significant main effect of sound volume on pupil dilation (F(2, 2504) = 51.6, p < 0.001), whereby both low (b = 0.07, p < 0.001) and high volume (b = 0.09, p < 0.001) induced larger pupil dilation compared to silent trials, and high volume induced larger pupil dilation compared to low volume (b = 0.02, p = 0.007). Results are shown in Fig. 2.

In terms of habituation, we found no significant effect of stimuli repetition on pupil dilation, but descriptively, the effect was in the expected direction (b =  − 0.01, F(1, 2504) = 3.22, p = 0.07).

### Visual orienting speed: first look latency

**Figure 3:**
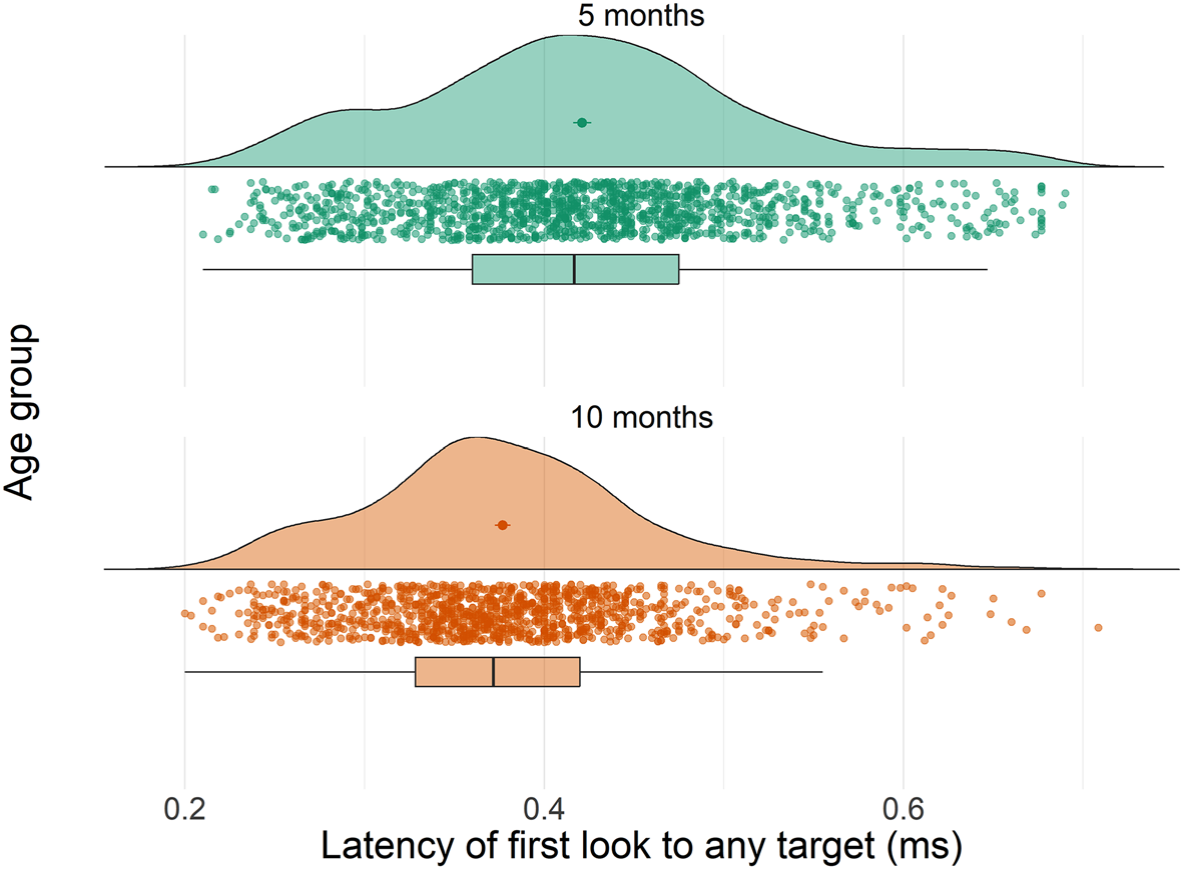
Developmental effect on visual orienting speed, indexed by latency of first look to any image in the visual array (in milliseconds). Data points in the figure show individual trial data while mean and standard error for each condition are shown in the clouds, and boxplots (with 25% to 75% whiskers) are reported below.

We found no significant effect of sound content (F(2, 2476) = 0.79, p = 0.45), sound volume (F(2, 2476) = 1.58, p = 0.21) or any repetition effect on latency of first look to any AOI (F(1, 2476) = 0.003, p = 0.96). Older infants had significantly faster looks to any AOI than younger infants (b =  − 0.09, F(1, 46.2) = 14.7, p < 0.001). Results are shown in Fig. 3.

However, we also found a significant interaction effect of age group and the inter-stimulus interval (ISI) on first look latency, showing a moderation by age on the ISI association with first look latency (F(1, 2489) = 6.91, p = 0.009). In particular, post-hoc tests between age groups at reference ISI levels (minimum 80 ms, average 230 ms, and maximum 400 ms) showed that age differences in first look latencies decrease for longer ISI. Specifically, we found 10-month-old vs. 5-month-old difference in latency equal to 0.125 s (standard error = 0.03; p < 0.001) for ISI = 80 ms; 0.09 s (standard error = 0.02; p < 0.001) for ISI = 230 ms; and 0.06 s (standard error = 0.03; p = 0.05) for ISI = 400 ms. This highlights the relevance of appropriate calibration of ISI for longitudinal comparisons.

### Social orienting: first look to face

**Figure 4:**
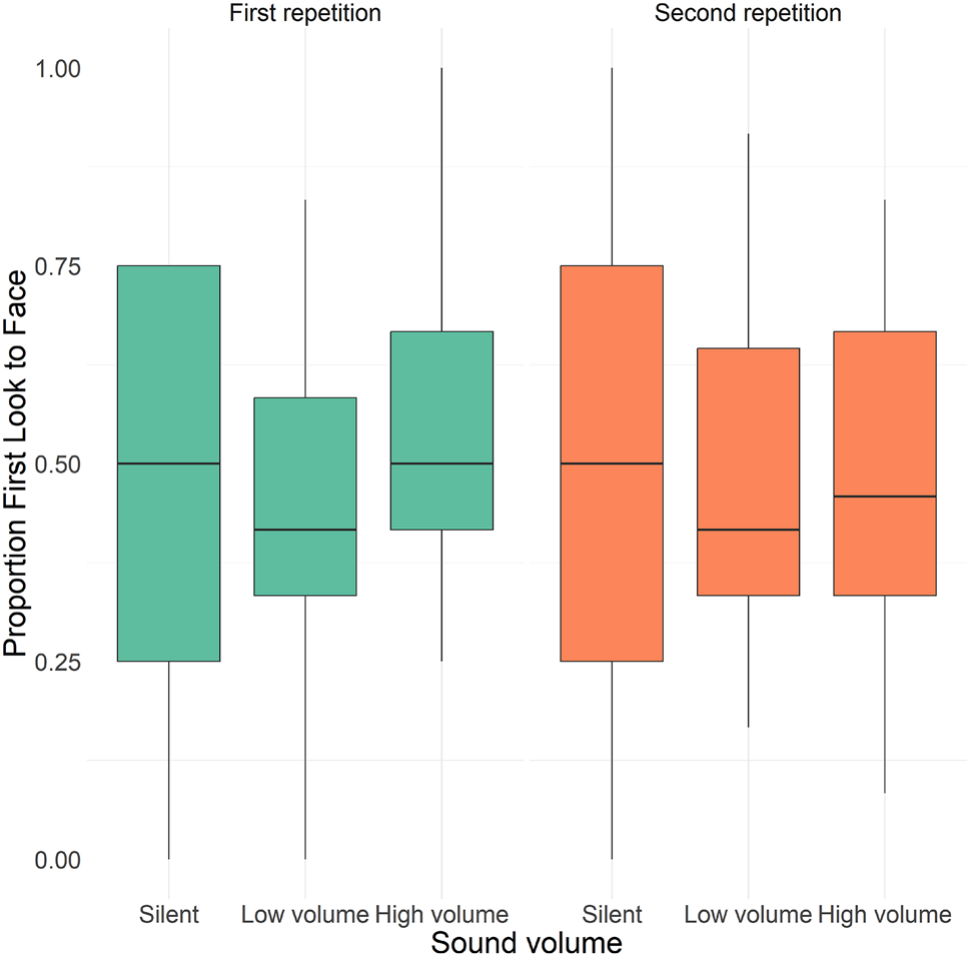
Effect of volume level of the auditory cue on target selection, shown as proportion of first look to the target (i.e., face) for each volume level condition by trial repetition.

We found a significant interaction effect of sound volume and repetition on target selection (i.e., first look to the face; χ^2^(5) = 13.0, p = 0.024). This effect was moderated by trial repetition, with more likely first looks to faces after sound cues in the high-volume condition compared to the low volume (marginal contrast = 0.44, SE = 0.13, p = 0.0015) in the first trial occurrence, but not when trials were repeated (or in the high-volume vs. silent, or low-volume vs. silent conditions; p*s* > 0.4). Age also had a significant main effect on likelihood of first look to the face, with significantly higher target selection at 10 months (b = 0.94, SE = 0.14, χ^2^(1) = 33.2, p < 0.001). We found no significant main effects of sound content (χ^2^(2) = 1.51, p = 0.47). Results are shown in Fig. 4.

### Relation between pupil dilation and visual orienting

**Figure 5:**
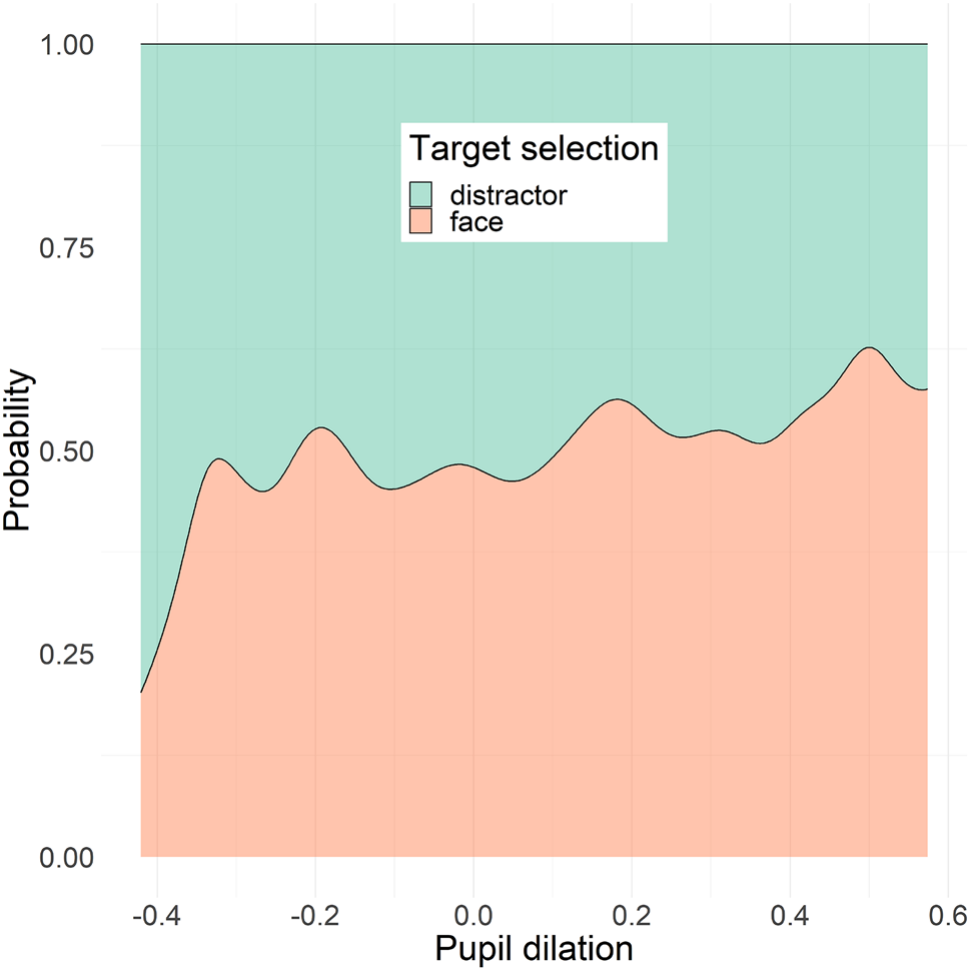
Effect of pupil dilation on target selection, shown as probability distribution of first gaze shift to the target (i.e., face) vs. another element in the visual array (i.e., else) by pupil dilation.

Average amplitude of pupil dilation response to sounds was significantly associated with target selection (i.e., first look to the face; b = 0.09, χ^2^(1) = 4.28, p = 0.039). We tested a quadratic relation between average pupil dilation and first look to the target (as planned in our pre-registered analyses: https://osf.io/wds3g), but it did not provide a better fit compared to the linear model (χ^2^(1) = 0.16, p = 0.69). Overall, our findings indicate a positive linear relation linking larger pupil dilation to increased likelihood of making the first look towards the face stimulus. This association was not moderated by effects of sound content (χ^2^(4) = 1.70, p = 0.79), volume (χ^2^(4) = 6.63, p = 0.16), or repetition (χ^2^(2) = 0.17, p = 0.92). Results are shown in Fig. 5.

**Figure 6:**
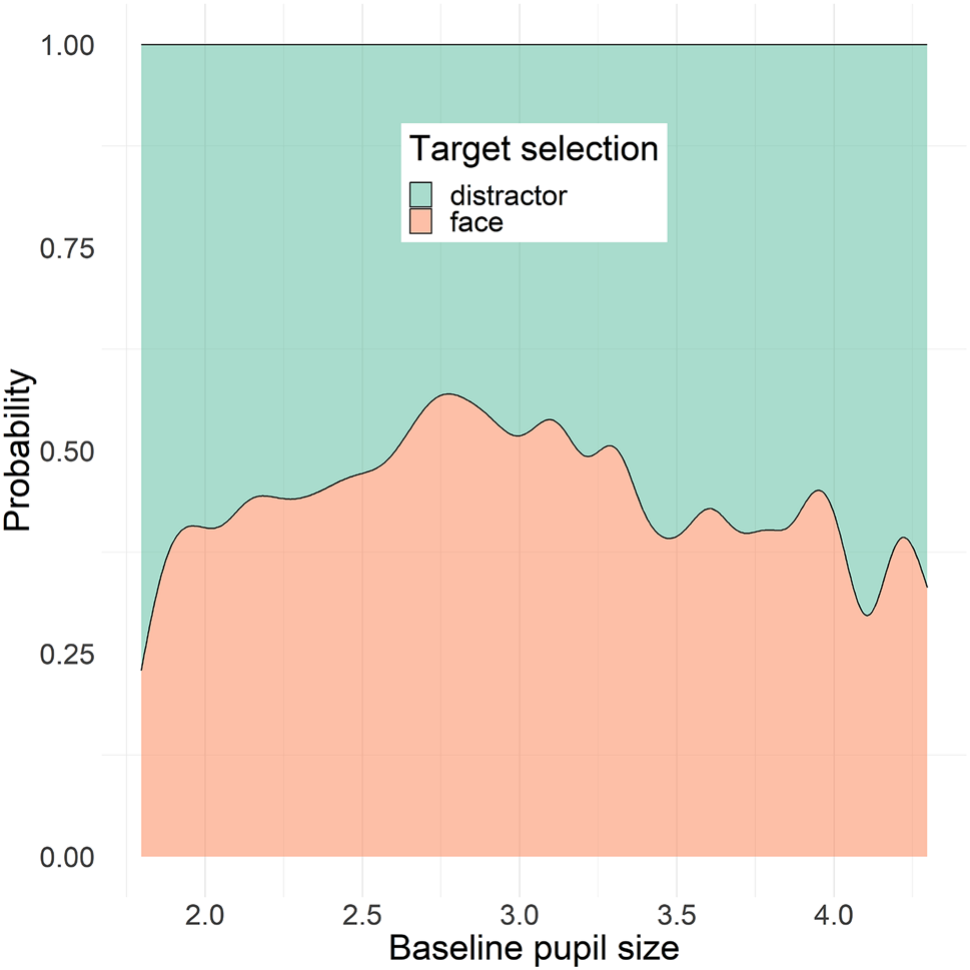
Effect of baseline pupil size (mm) on target selection, shown as probability distribution of first gaze shift to the target (i.e., face) vs. another element in the visual array (i.e., else) by pupil size.

To test whether the finding on the association between pupil dilation and target selection performance was due to differences between tonic and phasic modes of variation in pupil size, we performed an exploratory analysis testing the same model described above but using as independent variable baseline pupil size, measured as the average pupil size during the 200 ms before stimulus onset (see “Methods” section), instead of pupil dilation. Average baseline pupil size showed a quadratic association with target selection (b = − 1.0, SE = 0.5, χ^2^(1) = 4.04, p = 0.045), which was, however, not significant when accounting for age, proportion looking time on screen and inter-stimulus-interval covariates in the model (χ^2^(1) = 3.01, p = 0.08). Overall, our findings indicate an inverted U-shaped relation between baseline pupil size and likelihood of making the first look towards the face stimulus. Results are shown in Fig. 6.

**Figure 7:**
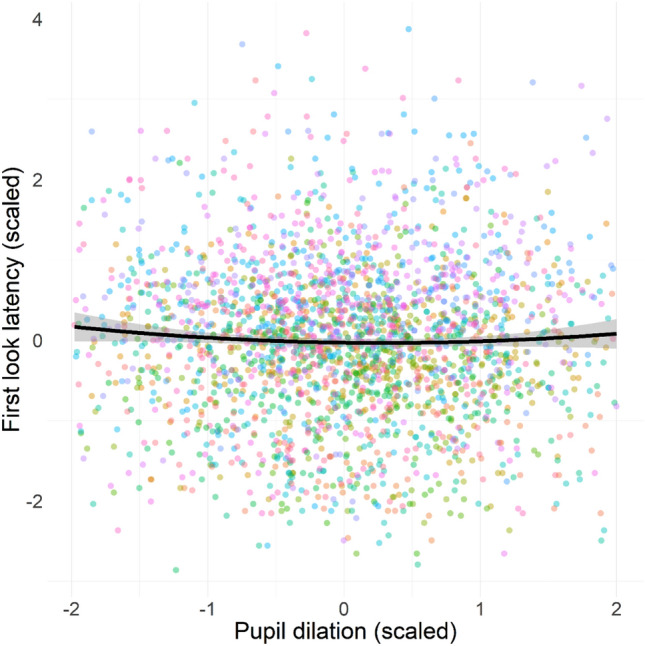
Effect of pupil dilation (standardized) on visual orienting speed (standardized), indexed by latency of first gaze shift to any image in the visual array. Individual-level trial data for each participant are shown by data points in different colours, while 95% confidence interval on the regression line is shown as shaded area.

While we did not find significant evidence for a quadratic relation between pupil dilation and target selection, there was a significant quadratic relation between pupil dilation and latency of first look to any AOI (b = 0.01, F(1, 2489) = 3.87, p = 0.049, tested within robust ranges to minimize edge effects on the quadratic term). Interaction effects with sound content (F(2, 2474) = 0.43, p = 0.65), volume (F(2, 2474) = 0.31, p = 0.73), and repetition (F(1, 2474) = 0.20, p = 0.65) did not contribute to the association between pupil dilation and visual orienting speed. This indicates a U-shaped relation whereby speed of visual orienting appears to be faster at intermediate levels of phasic arousal, as indexed by pupil dilation. Results are shown in Fig. 7.

## Discussion

The main aim of this study was to investigate the effect of experimental manipulation on induced arousal and how this influences the association between induced arousal level and visual attention at different developmental stages in infancy. Our findings extend previous work focusing on: (1) the modulatory role of social significance and volume of the auditory stimuli used to elicit phasic alertness; (2) habituation effects in phasic alertness; (3) the influence of arousal level on social attention (i.e., visual orienting to the face); (4) developmental effects on the modulation of phasic arousal and its relation with visual orienting to faces in infancy.

All auditory cues employed in this study to induce phasic alertness elicited significant pupil dilation of different amplitudes, showing that the alerting effects of sounds can be measured through pupillary responses already in the first year of life, as also shown by our previous work with infants^11,12^. Results from the different sound manipulations showed a separation between physical and content-related features of the alerting cue affecting arousal and visual orienting. Manipulation of sound volume level had a significant effect on pupil dilation and target (i.e., face) selection, while sound content (social vs non-social) did not have an effect on measures of induced arousal and orienting. More specifically, louder sounds elicited higher levels of phasic arousal (i.e., pupil dilation) compared to sounds at lower volume level and silent trials, and increased visual orienting to the target (i.e., face) compared to sounds at lower volume level. However, the effect of sound volume condition on target selection was no longer significant when pupil dilation was added to the model as covariate. This suggests that phasic arousal may mediate increased orienting to the face after louder auditory cues, in line with our hypothesis on higher alerting to more deviant sounds^24^.

Previous studies have reported mixed results on the effect of social significance of auditory cues on alerting and orienting abilities. Our findings support recent work showing no significant differences in pupil response to a wider range of social vs. non-social sounds in 10-month-old infants^12^, and extend this to visual orienting responses. However, Kleberg et al.^11^ reported significant effects of complex (social) vs. basic (non-social) sounds on pupil dilation and visual orienting speed but not target selection in 6.5-month-old infants. It is possible that differences in the type of sounds may account for these differences across studies, as non-identical stimuli were used. In particular, in Kleberg et al., social sounds did not include infant-directed speech or any naturalistic sound, compared to our work. Furthermore, the main effect of sound type found by Kleberg et al. was interpreted in relation to stimulus complexity (complex vs. basic sound) rather than social content.

Previous work with adults and animals has shown a rapid habituation of pupil dilation response to repeated sounds, i.e., already from the second trial^22,25^. Our findings indicate that habituation of pupillary response to sounds is not as rapid in infants as it has been shown in adults. This negative finding might also be explained by higher individual variability in infants’ pupillary habituation to sounds compared to adults’ responses. The effect of repetition was descriptively in the expected direction, although not significant. However, habituation is dependent on the time interval between stimulus repetition and the number of intervening trials^22^; therefore, differences in findings might also be explained by differences in presentation timings between studies. Future work should manipulate inter-stimulus-intervals between different sound repetitions to further investigate habituation effects on pupillary response to sounds in infancy. Nevertheless, we observed a significant habituation effect on the association between sound volume and first look to the face target, which was not reflecting an analogous effect on pupil dilation. It is striking to observe a habituation effect on target selection already after one trial, while it was not significant on phasic arousal level. One possible explanation is that the increased arousal level due to the auditory cue condition enhanced habituation in orienting response to the repeated stimulus, as shown by the repetition effect being only significant for the high-volume vs. low-volume conditions^26,27^. This may also suggest that the pupillary response is a more sensitive, but likely less specific, index of sound detection compared to the associated visual orienting response.

We also found significant developmental effects on pupil dilation and visual orienting abilities, both in terms of speed of non-specific orienting response, as indexed by latency of first look to any element in the visual array, and target selection. In particular, we observed increased target selection (i.e., more first looks to the face) and faster orienting response at 10 months compared to 5 months. These findings are in line with developmental changes in gray and white matter underlying maturation of frontoparietal brain networks underlying saccadic behavior and control^28,29^.

Pupil size is commonly used within infant cognition research as a measure of arousal. Neurophysiological work in animal studies has linked pupil size to a complex pattern of brain circuitry and different neuromodulators, including the cholinergic BF-Ach system and the noradrenergic LC-NE system^30^, which is well-known for modulation of arousal level and alertness^1^ and overlap with orienting networks^31^. Our hypothesis of an inverted U-shaped relation between pupil dilation and target selection was based on previous empirical findings^11^. However, we did not find an inverted U-shaped relation but a positive linear relation between phasic arousal, as indexed by pupil dilation, and social orienting. Instead, we found indications for a U-shaped relation between arousal level and visual orienting speed. We cannot rule out the possibility that our experiment did not capture the full spectrum of arousal variability and therefore our findings showed only the part of the hypothesized curve, from low to average arousal levels. However, it is important to highlight that activity within the LC can be separated between two distinct modes: tonic, i.e., slow changes in baseline activity, and phasic activity, i.e., rapid changes in neuronal activity that are temporally linked to specific stimuli, tasks or decisions^32^. Like LC activity, pupil size also shows tonic and phasic fluctuations, which are linked to the LC activity modes. The Yerkes–Dodson curve linking arousal to cognitive performance in an inverted *U*-shaped relation has been linked to tonic LC activity^33–35^, which is in line with our post-hoc findings on the inverted U-shaped relation between baseline pupil size (used as an index of tonic arousal) and target selection.

Our findings showed improved task-relevant performance (i.e., visual orienting to the face) with higher phasic arousal level. This supports previous work in infants^36^ and adults^37,38^, and extends these findings to sound-induced alertness in relation to social orienting at a younger age. We interpret our findings in relation to the adaptive gain theory on the association between arousal and attention^1^, according to which phasic LC activity increases the gain of task-relevant inputs over noise, promoting exploitation of the current focus of attention over exploration of the environment. Phasic activity occurs about 100 ms after stimulus onset^3^ and is thought to regulate global switch in attention network activity and flexibility of the orienting response through noradrenergic projections to the temporoparietal junction^39–41^. While high level of tonic activity is not related to a specific task and is associated with more general exploration, distractibility and poorer task-related performance^1,42^, increased phasic activity is thought to facilitate adaptive behavioral responses to stimuli inducing a timely increase in target neuronal gain^32^. In our study, better social orienting abilities, as indexed by first look to the face among distractors, were likely to be mitigated by adaptive gain induced by increased phasic reactivity in LC-NE projections to brain areas that are relevant for social cognition, like anterior cingulate cortex, anterior insula and amygdala^43,44^. Future work should try to replicate these findings and possibly extend to more naturalistic social interactions to confirm modulatory effects of arousal level on social attention and social cognition more generally.

This study has limitations in the small sample size, the cross-sectional nature of the study, which limits our understanding of developmental changes in the mechanisms under investigation, and the relatively short inter-trial intervals which could confound pupil dilations across different trials. Nevertheless, a post-hoc power analysis on achieved sensitivity showed that with a sample of 46 infants across 2 age groups and a repeated-measure design with 56 measure occurrence within-participant (i.e., trial-level data; r = 0.50 maximum correlation among repeated pupillary measures), this study had 80% statistical power to detect a within-between measure interaction effect with an error probability α = 0.05 and effect size f = 0.08 (critical F(55, 2420) = 1.34). This provides enough power to detect main and interaction effects with age in this study. Further work should attempt to replicate the observed findings in larger samples, and extend to a longitudinal design to understand developmental mechanisms. In relation to the length of inter-trial intervals, it is notable that the pupil dilation after sounds appears to return to baseline rather quickly (after each trial, approximately 3.5 s), indicating that the relatively short inter-trial intervals may be used in this context (see Fig. S1). Furthermore, as operationalized here, fluctuations in tonic pupil size are evaluated on a 3.5 s time-frame, which is shorter than minute-long timescales used to appreciate tonic activity^45^, but justified by time restrictions in experimental trials with infants, and anyway longer than what reported before^46^. One surprising finding from our study is the quadratic relation whereby visual orienting to any target was faster for intermediate levels of arousal, but slower for high levels of phasic arousal. This is in contrast with theories on neuronal gain and improved adaptive performance for higher phasic arousal level. However, our hypothesis considers orienting to the face to be the relevant task in an experiment measuring spontaneous visual orienting, which is not clearly comparable to previous task-based work. Future work should investigate this relation further. Furthermore, it remains an important question for future studies to investigate the interaction between baseline pupil size and phasic pupil dilation in relation to attention across development, as well as the effect of length of the inter-stimulus interval between auditory cue and appearance of the visual array on visual orienting across development, which showed a significant differential influence on speed of orienting response between 5 and 10 months in our study. In particular, separate events (i.e., sound and visual stimulus) occurring at short time intervals might be perceived as simultaneously occurring multisensory events, and the critical length of inter-stimulus interval for perceptual discrimination of events might differ across development.

Overall, our findings highlight the utility of pupillary measures for investigating early development of attention systems in infancy and provide a novel contribution to the understanding of the interplay between phasic arousal and attention in infancy. In particular, we provide first evidence in line with a neuronal gain model for spontaneous social orienting early in infancy, extending previous task-based work at older age. Future work should investigate generalizability of our findings across a wider socioeconomic distribution and in other (e.g. non-western) cultures. Further work should also focus on replicating the observed associations between tonic pupil size and phasic dilation in the context of spontaneous behaviour and further explore conditions under which fast pupillary habituation may be observable in human infants (such as for high volume conditions). Such work could have implications for both our basic understanding of attention systems early in life and potentially for individual differences and neurodevelopmental conditions linked to sensory processing (e.g., autism).

## Methods

### Participants

We recruited 59 participants from 2 different age groups (n = 29.5-month-old infants, and n = 30.10-month-old infants) from a local database of families who had expressed interest to participate in developmental research. Initial exclusion criteria for this study were: visual or auditory impairment, other medical diagnoses that could affect the infant’s development, and premature birth (before 37 gestational weeks). All participants were born in Sweden and came from middle- to high-income families with a high-level education background (see Table 1). Sample distribution in terms of ethnicity and socioeconomic status is the result of voluntary participation in the study and does not reflect any specific sampling stratification on those metrics. This study received ethical approval by the regional research ethics committee in Stockholm, Sweden, and all procedures were performed in accordance with the 1964 Helsinki declaration and its later amendments. Informed consent was obtained from parents of all individual participants included in the study.

**Table 1:** Demographics.

	Overall	5-month-old	10-month-old
Number of participants	46	25	21
Sex (male)	27	13	14
Parental education			
Upper secondary school: 3 to 4 years	6	5	1
Tertiary education: up to 5 years	16	6	10
Tertiary education: more than 5 years	23	14	9
Family income			
30–40 k SEK	4	3	1
40–50 k SEK	5	4	1
50–60 k SEK	7	5	2
60–70 k SEK	8	4	4
70–80 k SEK	8	3	5
80–90 k SEK	8	4	4
> 100 k SEK	5	2	3
Number of valid trials***	50.0 (5.18)	48.5 (5.54)	51.9 (4.13)

Continuous data are reported as *mean (standard deviation)* across the entire sample and stratified by age*.*

***Before imputation.

One infant was excluded from data analysis due to technical problems during data acquisition, and 12 infants were excluded because they contributed too little valid data (below 70%; largely due to the infant being distracted, feeling uncomfortable with the stimuli or moving too much), leading to a final sample size of 46 infants (27 male; 25.5-month-old) for data analysis. There were no significant differences in sex distribution across the two different age groups (χ^2^(1) = 0.50, p = 0.48). For this sample, we report patterns of missing data not completely at random based on association of missingness with other variables (e.g. more boys among excluded participants (t(18) =  − 2.32; p = 0.03), and more 10-month-old infants were excluded (t(18) =  − 2.12; p = 0.048)). To deal with missing data, we performed multiple (n = 100) non-parametric imputation on trial-level data through a regression tree algorithm using the package *mice* in *R*^47^. Sample characteristics are reported in Table 1.

### Eye-tracking protocol

For the duration of the experiment, infants were seated on their caretaker’s lap at an approximate distance of 65 cm from the monitor. The exact distance was adjusted during the experimental session to ensure optimal signal to the eye-tracker. Room brightness was kept constant for all participants to control for luminance effects on pupillary metrics. The experimenter monitored the participant by direct observation (through a webcam) and by use of a separate screen that indicated participant gaze position. A 5-point calibration procedure was completed before the task began. Data were collected at 600 HZ using a Tobii Pro Spectrum eye-tracker^48^. Stimuli were presented using a custom-written Psychopy script^49^, publicly available on GitHub together with a detailed description of the experiment (see: https://github.com/datalowe/psychopy-infant-audiovis-et/).

The experimental paradigm was a modified version of the one used in a previous study^11^ and consisted of a spontaneous visual search task preceded by an auditory cue for a total of 56 trials. Before each trial, a silent animated attention grabber was presented in the centre of the screen to ensure that the infant looked at the center before the visual array appeared. Trial start was triggered once the eye tracker registered that participant gaze had been directed at the attention grabber for two fifths of the time during an interval of 500 ms. If the participant didn’t gaze at the attention grabber for 5 s, the experimenter was instructed to play an attention-grabbing sound. Once gaze was captured, the attention grabber disappeared and a social sound or non-social sound played, unless it was a silent trial. After 80–400 ms (exact duration randomly determined), an array of 4 images (one for each category described below) was displayed for 3000 ms (see Fig. 1). After the visual array had finished, an entirely white screen was displayed for 500 ms until trial end. A distraction video was presented after each block, showing people dancing with balls in their hands and music in the background, to keep infants engaged during the experiment.

Visual stimuli used throughout the experiment included 56 images for each of the categories: adult faces (of varying sex, age, and ethnicity^50^), man-made objects (e.g., hammer), natural objects (e.g., leaf) and computer-generated 3D geometric shapes (e.g., cube). Each trial included one image from each category, of size 9° × 9° (visual degrees), arranged in a cross-like shape where each image midpoint was at the same horizontal (12°) and vertical (6°) distance from monitor midpoint. Image position in the visual array was randomly selected for each trial and each participant, with some restrictions: (1) each image only appeared once during the experiment; (2) one image from each category was included in each trial; (3) images of the same category never appeared in the same position two trials in a row.

The auditory cue varied in terms of content: social sound (24 trials), non-social sound (24 trials), and silence (8 trials). Social sound stimuli consisted of infant-directed speech; specifically, the same 3 Swedish words (*hejsan*, which means *hi*; *oj*, which means *oh*; *boll*, which means *ball*) recorded by a female and a male voice (for a total of 6 sounds balanced across gender). Non-social sound stimuli consisted of 6 recordings of everyday objects: baby rattle, dice roll, door being closed, toy car rolling, phone message sound, and toy squeak. Sound durations ranged between 506 and 989 ms. Each sound had a sudden onset, reaching a relative peak within 50 ms, though absolute maximum amplitude was sometimes reached later. All sound files shared the same maximum amplitude. The auditory cue level was also manipulated in terms of volume level so that 28 trials (50%) were played at a high volume (70 dB), and 28 at a comparably lower volume (i.e., 30% lower than high volume). The volume output on the computer and speakers was set and measured to 70 dB throughout the experiment for the sounds at the maximum level (measured from the participant’s seat). Calibration sounds and distraction video sounds were played at the high volume.

**Figure 8:**
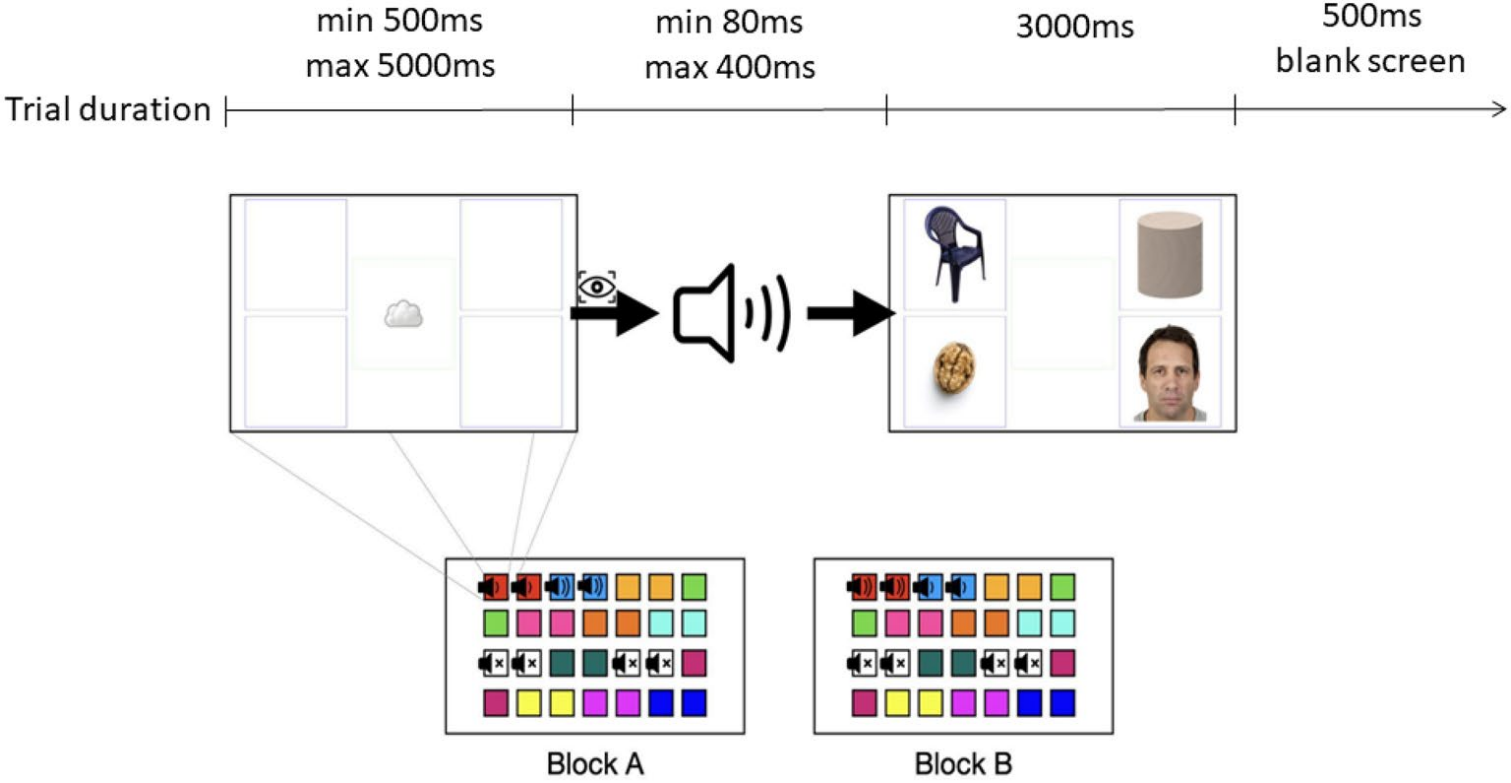
The experiment consisted of two blocks, each including 28 trials. Colours indicate different sounds, with white indicating silent trials. Sounds played at low volume in block A were played at high volume in block B, and vice versa. Each trial started with an attention grabber phase. Once gaze was captured, a sound started playing, and thereafter an array of images was displayed on the screen. Note that rectangles around visual stimuli denote regions of interest and were not present on participant screen. Face images used in the visual array, like the one shown in the present figure, were taken from a free resource database for scientific research, the Chicago Face Database^50^.

Trials always came in pairs, where both trials in a pair played the same sound at the same volume to account for the *Order* condition. Trial pairs in turn were arranged in two blocks (A and B), where each block consisted of one trial pair for each sound (12 pairs; 24 trials), and two silent trial pairs, thus in total 14 pairs (28 trials) per block (see Fig. 8). The two blocks had the same sound sequence but different volumes, to account for the *Volume* condition. For example, if during block A the baby rattle sound was played at low volume for two trials (one trial pair), then in block B the rattle sound would be played at a high volume for two trials (one trial pair). This ensured that the same sound was played in both volumes. Each block contained all sounds presented to the participant, balanced for social vs. non-social content, and only differed in volume level for the sounds presented in each block. Block sound sequence and volume was randomized for each participant.

### Pre-processing of eye-tracking data

#### Gaze

Gaze data was first linearly interpolated (over gaps in the data shorter than 150 ms) and averaged for the left and right eye. Areas of interest (AOIs) of 11° × 11° were defined around the four stimulus positions (see rectangles in Fig. 8) and binary vectors were computed for gaze in each AOI. Sum of time in each AOI (in seconds) was calculated, as well as the latency to enter the AOI for the first time from onset of the visual array. For the latter, only the first entry lasting at least 100 ms was considered (latency to enter an AOI was defined from the first accepted sample). In addition, proportion of raw missing data (before interpolation) in the entire trial and proportion of on-screen data during visual presentation (after interpolation) were computed.

Trials were rejected if: (1) interpolated gaze was not at the central attention-grabbing area for more than 40% of the 500 ms time-interval before sound onset; (2) interpolated gaze was not at all within the central attention-grabbing area during the 200 ms *before* visual onset; (3) interpolated gaze was missing or outside the screen during the 500 ms after visual onset, or was missing or outside the screen for more than 25% during the subsequent 500 ms (this was decided on not to exclude trials with a valid first gaze shift to an AOI, although the infant disengaged from stimuli quickly after); (4) gaze was not detected at any AOI (entry which lasted 100 ms or more) during the entire visual presentation; (5) latency to first AOI was below 200 ms or higher than 1 s. These criteria were defined based on previous studies^11,12^ and by looking at data from 4 participants (2 from each age group) at a pre-processing stage (see study pre-registration: https://osf.io/wds3g). Thresholds for latency validity (criterion 5) were decided based on the distribution of latencies to first AOI in all valid trials across all participants (n = 59 participants, n = 2710 trials), representing 1.2% and 0.5% of valid trials, respectively.

#### Pupil size

Pupil size data was first smoothed (using a moving average filter with 100 ms window), then averaged with a dynamic offset mean (i.e., taking into consideration the dynamic offset between the size of the two pupils in the gaps where one eye was missing^51^). Invalid samples (i.e., outside the 1.5–9 mm range, or either higher or lower than 3 standard deviations from the trial mean) were recoded into missing samples. Pupil samples when gaze was detected outside the screen were also recoded into missing samples. The pupil data was then linearly interpolated over gaps shorter than 150 ms.

Baseline pupil size was computed by taking the mean interpolated pupil size during the 200 ms preceding the sound onset (or the corresponding marker for silent trials). This is likely a period where participants provide valid data, given the contingency rule of the task; it is before the experimental manipulation of interest, and it is of reasonable duration^52^. Response pupil size was computed by taking the average interpolated pupil size during the first 2 s after the visual onset. This was decided based on the normalized pupil response across all trials of 4 preliminary participants (see Fig. S1), and is comparable with previous work^11,12^. Pupil dilation was computed by subtracting the average baseline pupil size from the average response pupil size.

In addition, proportion of raw missing data (before interpolation) in the entire trial and proportion of missing data during baseline and response (separately, after interpolation) were computed. Only valid trials for gaze data were considered for pupil measurements.

### Statistical analysis

The analysis plan, with aims and hypotheses, was pre-registered in Open Science Framework (OSF; see https://osf.io/wds3g) after data collection but prior to data analysis.

We used linear mixed models to model main and interaction effects of age and auditory cue conditions (content, volume, and repetition) on phasic arousal level (i.e., pupil dilation) and visual orienting (i.e., latency to first AOI as visual orienting speed, and first look to face as target selection) at a trial level while accounting for variance explained by random effects at the level of the individual infant^53^. Average amplitude of pupil dilation and latency of first look to any AOI were modelled as dependent variables in two separate models, respectively used as an index of phasic arousal level and visual orienting speed. Random effects were modelled on the intercept at the level of the individual infant, which is equivalent to a repeated measure approach on the trial data within participant. Models were fitted with a maximum likelihood approach and significance of fixed effects was tested by comparing models on single term deletions using likelihood-ratio tests (LRT) using the function *anova* in *R*. This approach has been shown to provide adequate *Type 1* error rates in the estimate of significant effects in linear mixed models^54^. Among fixed effects, we tested age (binary variable: 5 or 10 months), sex (binary variable), sound content (3-level categorical variable, contrast coded), sound volume (3-level categorical variable, contrast coded), repetition (binary variable), and measures of data quality (continuous variables: proportion of missing data per trial; length of inter-stimulus interval (i.e., time between sound cue and visual array onset; ISI); and number of valid trials per participant). Finally, we tested significance of interaction effects among the different auditory cue conditions; between the different auditory cue conditions and age; and with the inter-stimulus interval.

To investigate modulation of social orienting by different auditory cue conditions and age, target selection (here, first look towards the face image in the visual array) was modelled through generalized mixed models with a binomial distribution and a logit link function. Target selection was coded as a binary variable, with value 1 when the first look was on the face target, and 0 when the first look was in a different AOI. A similar approach was used for model selection, including individual random effects on the intercept and testing significance of fixed effects through model comparison based on LRTs.

Similarly, we tested the hypothesis that phasic arousal could mediate the effect of alerting sounds on visual orienting speed and target selection by modelling target selection and latency of first look to any target on average pupil dilation using mixed models.

Pre-processing of eye-tracking data was performed using custom made scripts in Matlab^55^ while statistical analyses were performed in *R*^56^ using scripts that are publicly available at https://github.com/brainhabit/pupil_popout.

### Supplementary Information

Supplementary Figure S1.

## Data Availability

The data necessary to reproduce the analyses presented here will be made available upon reasonable request to the corresponding author. Note that sharing of pseudonymized personal data will require a data processor agreement (DPA), according to the Swedish and EU law. The materials necessary to attempt to replicate the findings presented here are publicly available at https://github.com/datalowe/psychopy-infant-audiovis-et, while the analytic code is available at: https://github.com/brainhabit/pupil_popout.
