# Supplementary material for: Manipulation of phasic arousal by auditory cues is associated with subsequent changes in visual orienting to faces in infancy
Source: Sci Rep. 2023 Dec 12;13:22072. doi: 10.1038/s41598-023-49373-x (PMC10716513; doi:10.1038/s41598-023-49373-x)
Supplement: Supplementary file 1 — Supplementary Figure S1. [file 41598_2023_49373_MOESM1_ESM.pdf]

Supplementary Material

Manipulation of phasic arousal by auditory cues is associated with subsequent changes in visual orienting to faces in infancy.

Giorgia Bussu, Ana Maria Portugal, Lowe Wilsson, Johan Lundin Kleberg, Terje Falck-Ytter

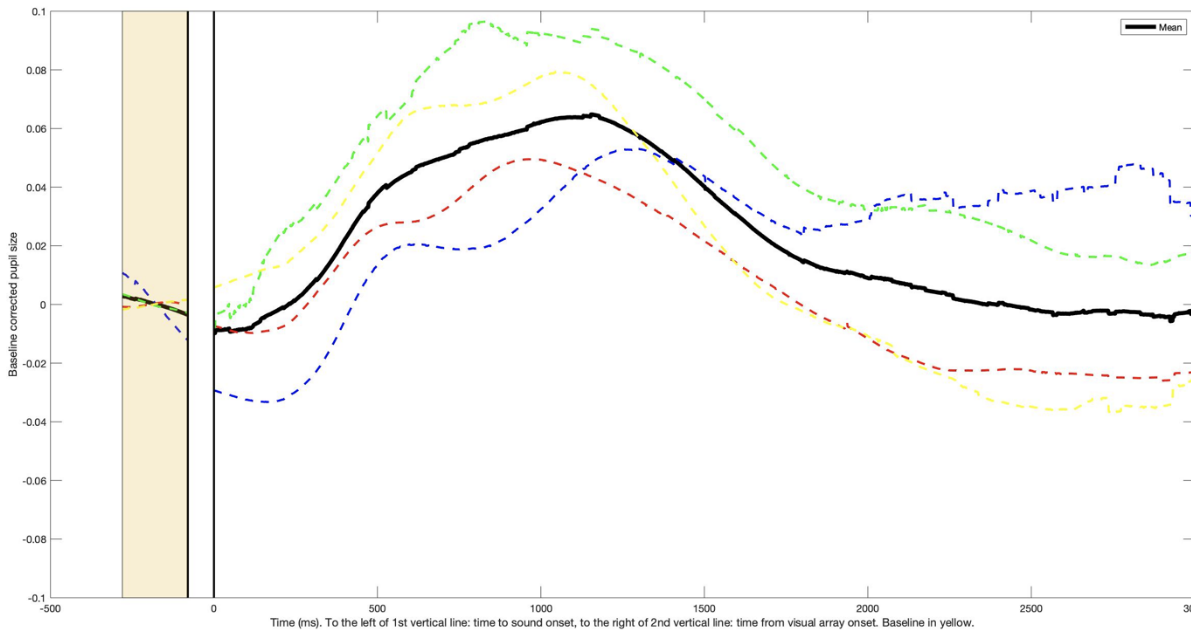

Figure S1. Time-course of pupil dilation. This figure is based on 4 preliminary participants.
